# Carbapenem resistant *Enterobacterales* in the United Arab Emirates: a retrospective analysis from 2010 to 2021

**DOI:** 10.3389/fpubh.2023.1244482

**Published:** 2023-12-07

**Authors:** Jens Thomsen, Najiba M. Abdulrazzaq, The UAE AMR Surveillance Consortium, Dean B. Everett, Godfred Antony Menezes, Abiola Senok, Carole Ayoub Moubareck

**Affiliations:** ^1^Abu Dhabi Public Health Center, Abu Dhabi, United Arab Emirates; ^2^Department of Pathology and Infectious Diseases, Khalifa University, Abu Dhabi, United Arab Emirates; ^3^Emirates Health Services Establishment, Dubai, United Arab Emirates; ^4^Biotechnology Research Center, Khalifa University, Abu Dhabi, United Arab Emirates; ^5^Infection Research Unit, Khalifa University, Abu Dhabi, United Arab Emirates; ^6^Department of Medical Microbiology and Immunology, Ras Al Khaimah Medical and Health Sciences University, Ras Al Khaimah, United Arab Emirates; ^7^College of Medicine, Mohammed Bin Rashid University of Medicine and Health Sciences, Dubai, United Arab Emirates; ^8^School of Dentistry, Cardiff University, Cardiff, United Kingdom; ^9^College of Natural and Health Sciences, Zayed University, Dubai, United Arab Emirates

**Keywords:** carbapenem-resistant *Enterobacterales*, surveillance, *Enterobacterales*, healthcare associated infections, antibiotics, antimicrobial resistance, UAE

## Abstract

**Background:**

Carbapenem-resistant *Enterobacterales* (CRE) are spreading in the United Arab Emirates (UAE) where their dissemination is facilitated by international travel, trade, and tourism. The objective of this study is to describe the longitudinal changes of CRE as reported by the national AMR surveillance system of the UAE.

**Methods:**

In this study, we retrospectively describe CRE isolated from 317 surveillance sites, including 87 hospitals and 230 centers/clinics from 2010 to 2021. The associated clinical, demographic, and microbiological characteristics are presented by relying on the UAE national AMR surveillance program. Data was analyzed using WHONET microbiology laboratory database software (http://www.whonet.org).

**Results:**

A total of 14,593 carbapenem resistant *Enterobacterales* were analyzed, of which 48.1% were carbapenem resistant *Klebsiella pneumoniae* (CRKp), 25.1% carbapenem resistant *Escherichia coli* (CREc), and 26.8% represented 72 other carbapenem resistant species. Carbapenem resistant strains were mostly associated with adults and isolated from urine samples (36.9% of CRKp and 66.6% of CREc) followed by respiratory samples (26.95% for CRKp) and soft tissue samples (19.5% for CRKp). Over the studied period carbapenem resistance rates remained high, especially in *K. pneumoniae*, and in 2021 were equivalent to 67.6% for imipenem, 76.2% for meropenem, and 91.6% for ertapenem. Nevertheless, there was a statistically significant decreasing trend for imipenem and meropenem resistance in *Klebsiella species* (*p* < 0.01) while the decrease in ertapenem resistance was non-significant. Concerning *E. coli*, there was a statistically significant decreasing trend for meropenem and imipenem resistance over the 12 years, while ertapenem resistance increased significantly with 83.8% of *E. coli* exhibiting ertapenem resistance in 2021. Resistance rates to ceftazidime and cefotaxime remained higher than 90% (in 2021) for CRKp and cefotaxime rates increased to 90.5% in 2021 for CREc. Starting 2014, resistance to colistin and tigecycline was observed in carbapenem resistant *Enterobacterales*. CRE were associated with a higher mortality (RR: 6.3), admission to ICU (RR 3.9), and increased length of stay (LOS; 10 excess inpatient days per CRE case).

**Conclusion:**

This study supports the need to monitor CRE in the UAE and draws attention to the significant increase of ertapenem resistance in *E. coli*. Future surveillance analysis should include a genetic description of carbapenem resistance to provide new strategies.

## 1 Introduction

Nowadays, carbapenem-resistant *Enterobacterales* (CRE) represent a serious health concern worldwide, causing a distressing burden on morbidity, mortality and healthcare costs, and contributing to the socio-economic and public health consequences of antimicrobial resistance (1, 2). Gram-negative, rod-shaped, facultatively anaerobic bacteria inhabiting the gastrointestinal tract, *Enterobacterales* (formerly *Enterobacteriaceae*) represent the largest group of bacterial pathogens in humans (3, 4). They are associated with a wide range of severe infections including septicemia, urinary tract infections (UTIs), intra-abdominal infections, and pneumonia, which can be community-acquired, hospital-acquired, or ventilator-associated (5–9). The widespread, empiric use of carbapenems as the most reliable antibiotics for the treatment of infections caused by extended-spectrum β-lactamase (ESBL)-producing *Enterobacterales* has driven the emergence of CRE, whose infections are more challenging to treat (10). According to the Centers for Disease Control and Prevention (CDC), CRE are defined as *Enterobacterales* strains that test resistant to at least one of the carbapenem antibiotics (ertapenem, meropenem, doripenem, or imipenem) or produce a carbapenemase (11). CRE acquire resistance to carbapenems via efflux pump overactivity, loss or mutation of outer membrane proteins, and/or carbapenemase production, the latter being the most prevalent mechanism (12, 13). With increasing incidence of infections caused by CRE and the lack of new, approved treatment modalities, such infections are associated with worse outcomes, lengthier hospitalizations, and increased costs compared to their susceptible counterparts (14). CRE continue to be labeled as critical priority pathogens by the World Health Organization (WHO), and the necessity for discovery, research, and development of new antibiotics targeting these pathogens remains an urgent need (15).

The global spread of CRE and changes in their epidemiology continue to evolve, inevitably complicating therapy and hampering effective antimicrobial stewardship and infection prevention and control programs (1, 16). In general, longitudinal studies of antibiotic susceptibility in a specific region over time allow identification of trends of resistance and emerging pathogens at national levels. Such routine surveillance is key for generating and establishing approaches to control antimicrobial resistance and guide informed therapy decisions (16), and appears critical as far as CRE are concerned (17). The trends obtained will detect either a rise in CRE prevalence (18, 19), thus revisiting and improving the current infection control strategies, or its decline (20), thus reinforcing the possible beneficial factors. The United Arab Emirates (UAE), a thriving hub for international travel, trade, tourism and medical services, has been susceptible to CRE spread, like many other countries in the Arabian Peninsula (21). Currently, the country hosts a population of nearly 10 million people of which approximately 1 million are Emirati citizens, and the rest are expatriates from various nationalities. The majority of this population resides in Abu Dhabi and Dubai, the two biggest Emirates of the seven that form the UAE (22).

Previous data have described the epidemiology and resistance patterns of CRE from the UAE, the latest of which being the study by Pál et al. (17), which compared CRE collected between 2009 and 2015 to those collected between 2018 and 2019 in the Emirate of Abu Dhabi. The study revealed that highly resistant *Klebsiella pneumoniae* clones started dominating the area since 2009, severely impacting the overall antibiotic resistance patterns, including those of colistin and tigecycline. Moreover, a recent surveillance of CRE carried out over 9 months in 15 Emirati hospitals showed around 100% non-susceptibility to ertapenem and 80% non-susceptibility to each of imipenem and meropenem (23). Likewise, resistance rates of 100% to ertapenem, 21% to imipenem, and 17% meropenem were observed in a collection of *Enterobacterales* in an epidemiological investigation from Dubai (24). Smaller scale investigations of CRE in the UAE also reported clusters of NDM-1-producing *Enterobacterales* (25), and more recently of *K. pneumoniae* with OXA-181/NDM-5 carbapenemases (26). The accumulation of such body of evidence supports the notion that timely, focused, and systematic, surveillance could offer a possible guidance to health authorities to mitigate the countrywide progress of the CRE epidemic. As such, it is imperative to address the current gap in literature regarding the spread of CRE infections and their resistance trends over the years, especially given the multicultural, heterogeneous, and diverse nature of the UAE population.

The objective of the current study is to describe the characteristics and longitudinal changes in CRE resistance levels and trends as reported by the national AMR surveillance system spanning all the seven emirates of the UAE, in order to assess the nationwide status of the CRE epidemic. It represents the first documentation of changes in CRE isolated from UAE medical centers over a period of 12 years, from 2010 to 2021.

## 2 Materials and methods

### 2.1 Study design and data source

A multi-institutional retrospective observational study was conducted between 2010 and 2021 in the UAE using data extracted from the WHONET microbiology laboratory database software (www.whonet.org) supported by the Global AMR Surveillance System protocol (GLASS, World Health Organization). Data was generated, collected, cleaned, and analyzed through the UAE national AMR Surveillance programs as described by Thomsen et al. (27).

### 2.2 Identification and enrollment of national AMR surveillance sites

Starting 2010, UAE healthcare institutions were enrolled as AMR surveillance sites into the UAE national AMR surveillance program based on epidemiological needs assessment, readiness, and willingness of facilities to participate, availability of high-quality electronic AMR data, lab accreditation status, and qualification of staff. Hospitals, centers, and clinics representing all seven emirates of the UAE joined the AMR surveillance network gradually over the years.

### 2.3 Bacterial population and variables of the study

All *Enterobacterales* isolated from clinical samples by medical professionals in the national AMR surveillance sites were part of this surveillance analysis from January 2010 to December 2021. Repeat isolates were marked and only the first isolate was included for each patient per year.

The associated patient demographic information, clinical data, and microbiologic laboratory results were extracted from the national WHONET laboratory database software. The demographic variables included age, sex, nationality, clinical variables revealed the type of facility reporting the isolate (hospital/center/clinic), patient location, location type, specimen collection date, types of infection/specimen source, and microbiology variables revealed types of organism and antibiotic susceptibility testing results. The infection was considered to originate outside the center for outpatients or those presenting with the infection at the emergency department.

### 2.4 Bacterial identification

Bacterial identification was performed at the national AMR surveillance sites by medical professionals. The participating centers used at least one commercial, automated system for identification of bacteria, including VITEK^®^ (BioMérieux SA, Craponne, France), BD Phoenix™ (Becton Dickinson, New Jersey, USA) and MicroScan (Beckman Coulter, Brea, CA, USA). Only one lab relied on manual systems like API^®^ (Analytical Profile Index. BioMérieux SA, Craponne, France) solely for identification.

### 2.5 Antimicrobial susceptibility testing

Antimicrobial susceptibility testing was performed at the National AMR surveillance sites using at least one commercial, automated system for routine antimicrobial susceptibility testing. Only two laboratories used manual testing methods (disc diffusion/Kirby Bauer). All labs followed CLSI guidelines for antimicrobial susceptibility testing of bacteria (CLSI-M100) (28). The EUCAST guidelines were used for interpretation of tigecycline results (29). Unusual antibiotic susceptibility testing results were confirmed locally. To assess the multidrug-resistant (MDR) phenotype of the isolates, a slightly modified version of the standard definition by Magiorakos et al. (30) was used.

Strains of CRE were defined as *Enterobacterales* species such as *K. pneumoniae, E. coli, Klebsiella oxytoca, Enterobacter cloacae*, and *Enterobacter aerogenes* and others that are resistant to at least one carbapenem antibiotic or produce a carbapenemase enzyme. *Proteus* spp., *Morganella* spp., and *Providencia* spp. that have intrinsic elevated minimum inhibitory concentrations to imipenem but are susceptible to ertapenem and/or meropenem were not counted. Repeat isolates were marked and only the first ones expressing any distinct carbapenem resistance mechanisms were included for each patient during the surveillance period (2010–2021).

### 2.6 Statistical tests

Statistical significance of temporal trends for antimicrobial resistance percentages was calculated if data from at least 5 years was available. If fewer than 30 isolates per year were reported, or data was not available for all years within the considered period, trend analysis was not conducted. Statistical significance of trends is expressed as a *p*-value, calculated by a Chi-square for trend test (extended Mantel-Haenszel), using SPSS or Epi Info™. For testing the statistical significance of the difference for mortality and ICU admission a Chi^2^-test was used. For testing the statistical significance of the difference for length of stay (LOS), the weighted log-rank survival analysis was used. This was done to take care of differences in sample size between the groups. A *p* < 0.05 was considered statistically significant.

## 3 Results

### 3.1 Distribution of reporting sites for national AMR surveillance

**Figure 1:**
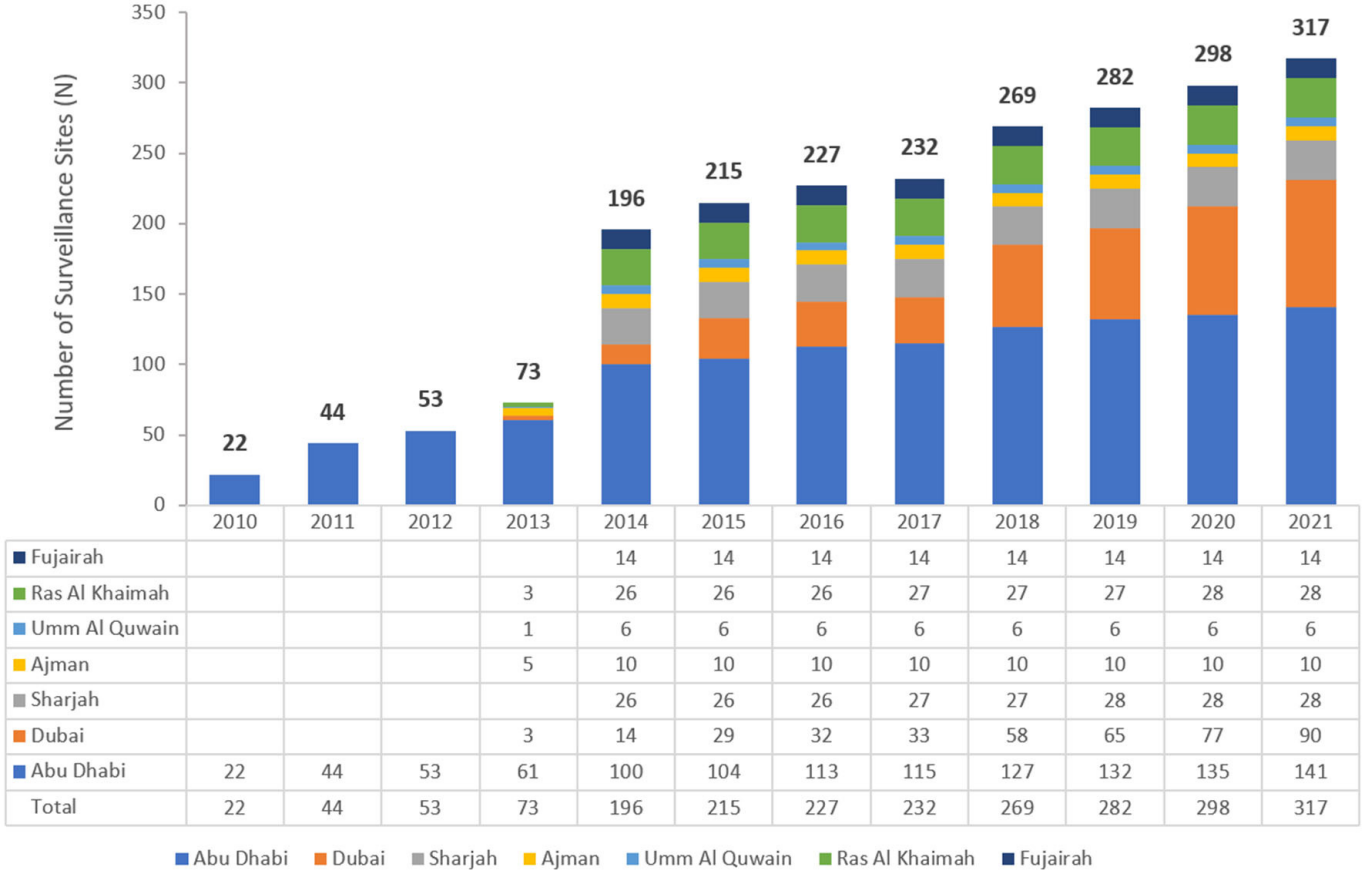
Number of AMR Surveillance sites by Emirate over the years of the surveillance period (2010–2021) in the UAE.

The UAE national AMR surveillance program was in 2010 in the Abu Dhabi Emirate with six hospitals and 16 centers/clinics enrolled as AMR surveillance sites. Additional sites were recruited over the years, starting with 22 participating sites located only in the Emirate of Abu Dhabi in 2010, which is the first year during which the study was initiated, and reaching in 2021 a total of 317 surveillance sites, including 87 hospitals and 230 centers/clinics and representing all seven emirates of the country. Figure 1 represents the distribution of reporting sites for National AMR Surveillance from 2010 to 2021.

### 3.2 Bacterial population and trend of carbapenem resistance over the years

**Figure 2:**
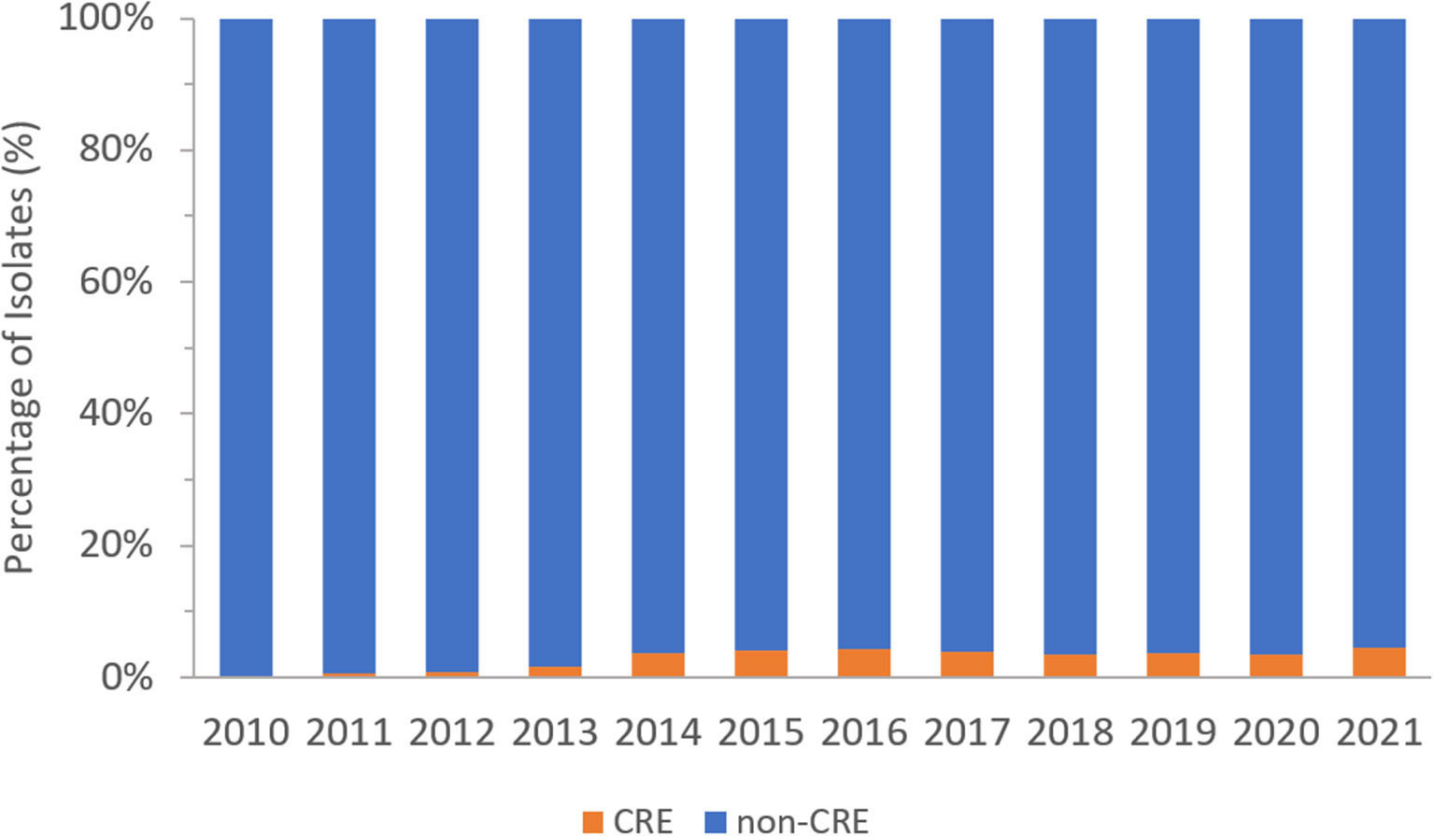
Percentage of CRE and non-CRE isolates per year over the surveillance period (2010–2021).

From 2010 to 2021, a total of 381,535 non-repetitive *Enterobacterales* were included in the analysis of which 14,593 (3.8%) were carbapenem resistant (CRE), representing 74 different species. Figure 2 represents the percentage of CRE and non-CRE isolates per year.

**Figure 3:**
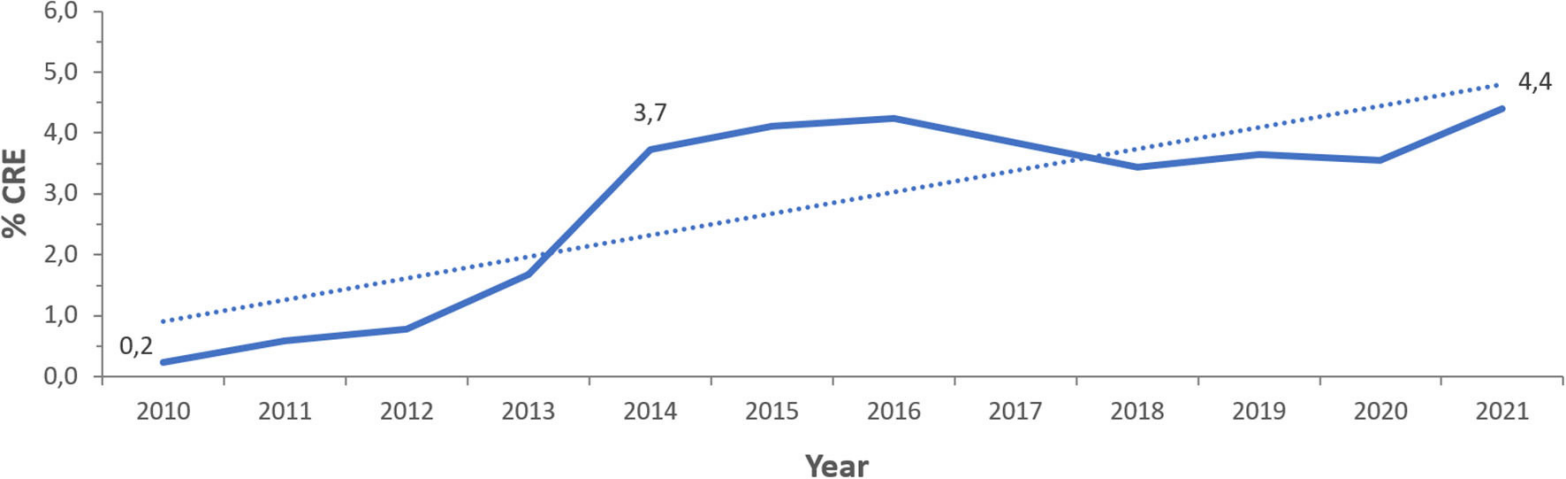
Time trends of CRE prevalence (% CRE) in the UAE over the surveillance period (2010–2021).

Figure 3 represents the prevalence of CRE calculated per year during the 12 years of the study. A gradual rise in this prevalence was seen from 2010 (0.2%) and for 4 consecutive years until 2014 (3.7%). Starting 2014, CRE prevalence was oscillating between 3.4 and 4.2%, with a steady decrease between 2016 and 2020, then showing a tendency to increase noted in 2021. The overall prevalence of CRE over the 12 years of surveillance averages at 3.8%.

### 3.3 Species distribution of CRE

**Figure 4:**
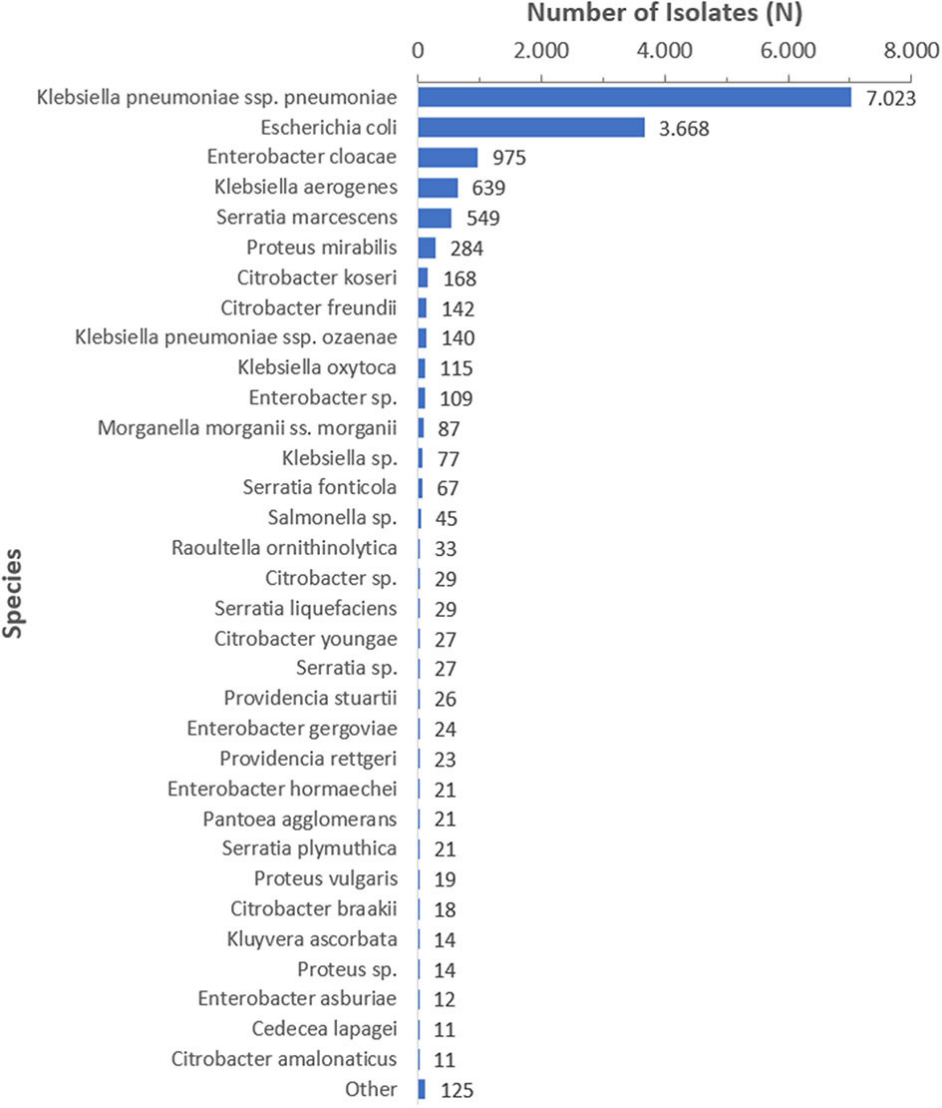
CRE species distribution over the surveillance period (2010–2021), by species (*N* = 14,593), species is shown for *n* ≥ 10.

Among the 14,593 carbapenem resistant *Enterobacterales* isolates analyzed, 7,023 (48.1%) were carbapenem resistant *K. pneumoniae* (CRKp), 3,668 (25.1%) were carbapenem resistant *Escherichia coli* (CREc), and the remaining 3,902 (26.8%) isolates represented 72 other species. The CRE species distribution over the surveillance period is shown for species with at least 10 isolates in Figure 4.

### 3.4 Distribution of CRE by age, gender, nationality, and emirate

Carbapenem resistant strains were mostly associated to infections in adults (89.6% of patients) with an average of 93.7 and 85.5% of infections caused, respectively, by CRKp and CREc in that population group. The number of CRE isolates recovered from patients below 19 years increased from two isolates in the first year of the study to 265 for CRKp and 373 for CREc in the last year.

The most commonly isolated species of CRE being CRKp, looking into features of these isolates revealed they were mostly recovered from male patients (57%). Patients were of unknown nationality (47.9%), although 16.3% of the patients were Emirati citizens. The most frequent Emirate for isolation of CRKp was Abu Dhabi (31.8%). Most patients developing infections due to CRKp were detected in clinical settings (83.9%) and were enrolled in general medical wards (48.1%) followed by ICUs (30.1%) and critical care units (1.2%). A proportion of 20.5% of studied isolates originated in outpatient basis, being recovered either in the community or in emergency departments.

Following CRKp, the second most frequent CRE species was CREc, commonly isolated from females (65.2%), of unknown nationality (44.4%), and in the Emirate of Abu Dhabi (43.4%). Most patients developing infections due to CREc were detected in clinical settings (50%) and were enrolled in general medical wards (31.8%) followed by ICUs (11%) and critical care units (0.2%). A proportion of 57% of studied isolates originated in outpatient basis, being recovered either in the community or in emergency departments.

### 3.5 Mortality rate

A subgroup analysis including the nine clinical institutions that reported mortality was performed. In these institutions, a total of 101,762 patients were associated with non-CRE of whom 3,717 patients died (mortality rate: 3.65%), while a total of 1,824 were associated with CRE, of whom 389 patients died (mortality rate: 21.33%). The difference in mortality between CRE patients and non-CRE patients is statistically significant (RR 6.31, 95% C.I. 5.74, 6.93, *p* < 0.01).

### 3.6 Admission to intensive care unit (ICU)

A total of 249,844 patients were associated with non-CRE of whom 13,567 patients were admitted to ICU (ICU admission rate is 5.43%), while a total of 10,011 patients were associated with CRE, of whom 2,142 patients were admitted to ICU (ICU admission rate: 21.40%). The difference in ICU admission rate is statistically significant (RR 3.94, 95% C.I. 3.78, 4.11, *p* < 0.01).

### 3.7 Length of stay (LOS)

A subgroup analysis including those patients for whom the date of admission as well as the date of discharge was known was performed (*N* = 34,195). For those patients who were associated with non-CRE (*n* = 33,462) the median length of stay was 7.0 days, while for those patients who were associated with CRE (*n* = 733) the median length of stay was 17.0 days, equivalent to 7,330 excess days of hospitalization. The difference in length of stay (LOS) was equal to 10 days and was statistically highly significant (*p* < 0.001).

After applying the above-mentioned difference in the LOS on the total number of patients associated with CRE (*n* = 14,593) during the whole observation period (2010–2021), a total of 145,930 excess days of hospitalization is estimated attributable to CRE. For the year 2021 only (*n* = 3,448 CRE cases), a total of 34,480 excess hospitalization days is estimated attributable to CRE.

### 3.8 Distribution of carbapenem resistance among the different clinical sample types

Carbapenem resistant strains were mostly isolated from urine samples (36.85% of CRKp and 66.55% of CREc) followed by sputum samples (26.95% of CRKp and 6.35% of CREc) and soft tissue samples (19.52% of CRKp and 13.79 % of CREc) as described in Tables 1, 2.

**Table 1:** Number and percentage of CRKp isolated during the study by clinical specimen type.

**Sample type**	**Number of CRKp**	**Percentage**
Urine	2,588	36.85
Respiratory	1,893	26.95
Soft tissue	1,371	19.52
Blood	535	7.62
Stool	165	2.35
Genital	66	0.94
Unknown/Other	405	5.77
Grand total	**7,023**	**100.00**

Bold values are highlighting the total (row sum), in comparison to the other numbers.

**Table 2:** Number and percentage of CREc isolated during the study by clinical specimen type.

**Sample type**	**Number of CREc**	**Percentage**
Urine	2,441	66.55
Soft tissue	506	13.79
Respiratory	233	6.35
Blood	148	4.03
Genital	90	2.45
Stool	36	0.98
Unknown/Other	214	5.83
Grand total	**3,668**	**100.00**

Bold values are highlighting the total (row sum), in comparison to the other numbers.

### 3.9 Trend of antimicrobial susceptibility profiles of CRE

**Figure 5:**
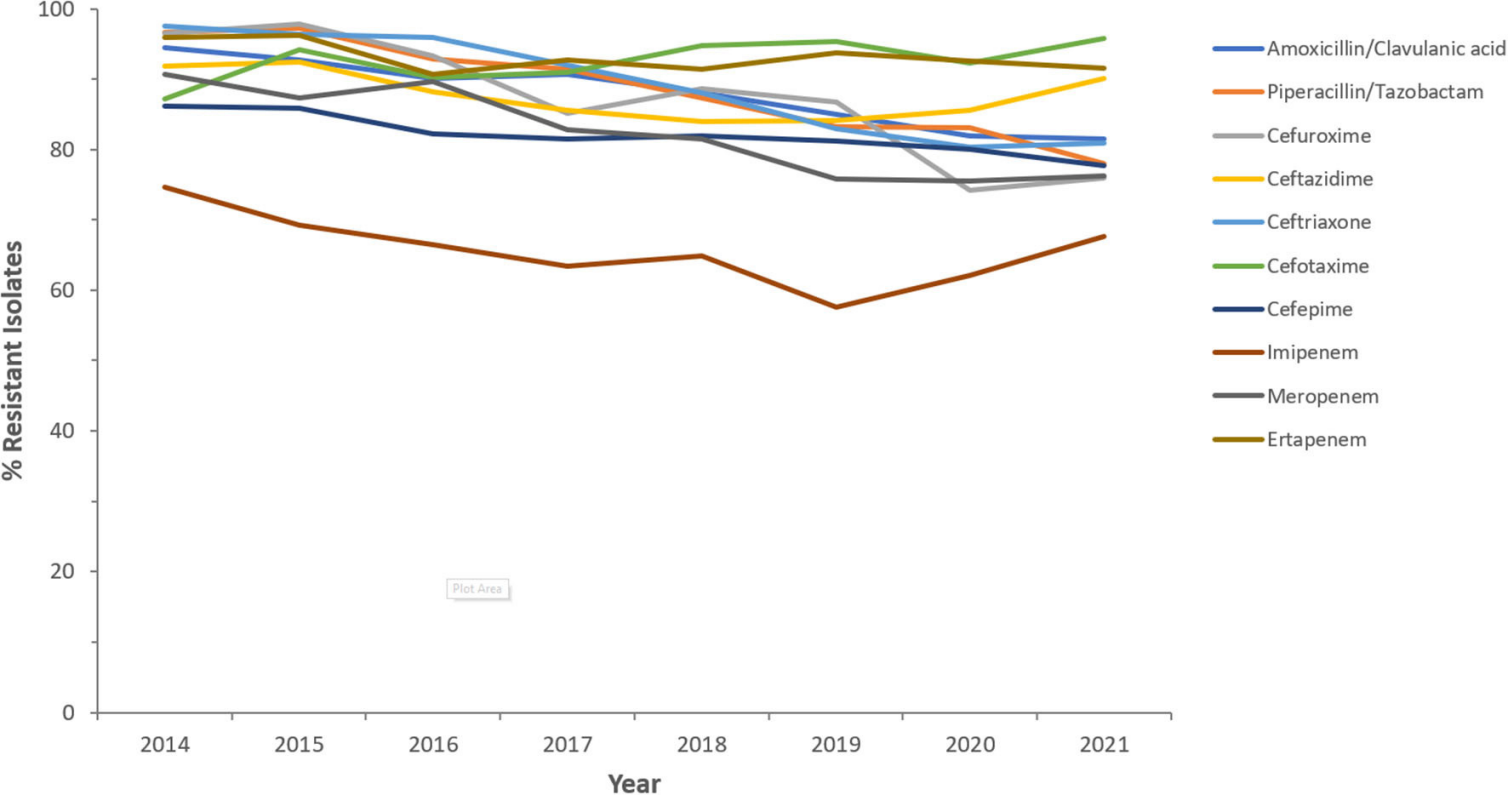
Resistance patterns (%R) to antibiotics of the β-lactam group among CRKp for the period between 2014 and 2021. The graph shows a selected period due to small number of participating centers prior to 2014.

**Figure 6:**
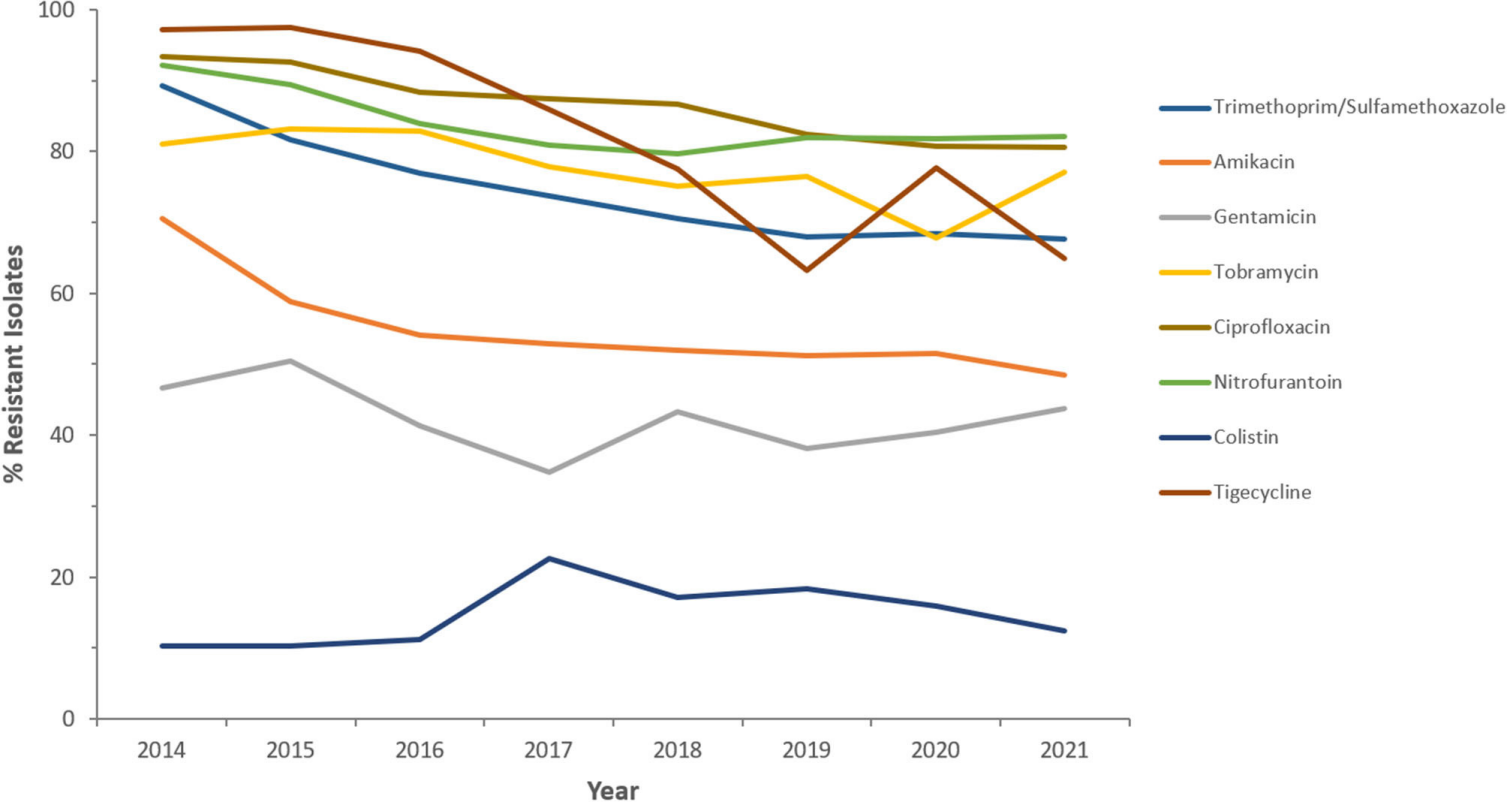
Resistance patterns (%R) to non β-lactam antibiotics among CRKp for the period between 2014 and 2021. The graph shows a selected period due to small number of participating centers prior to 2014.

**Figure 7:**
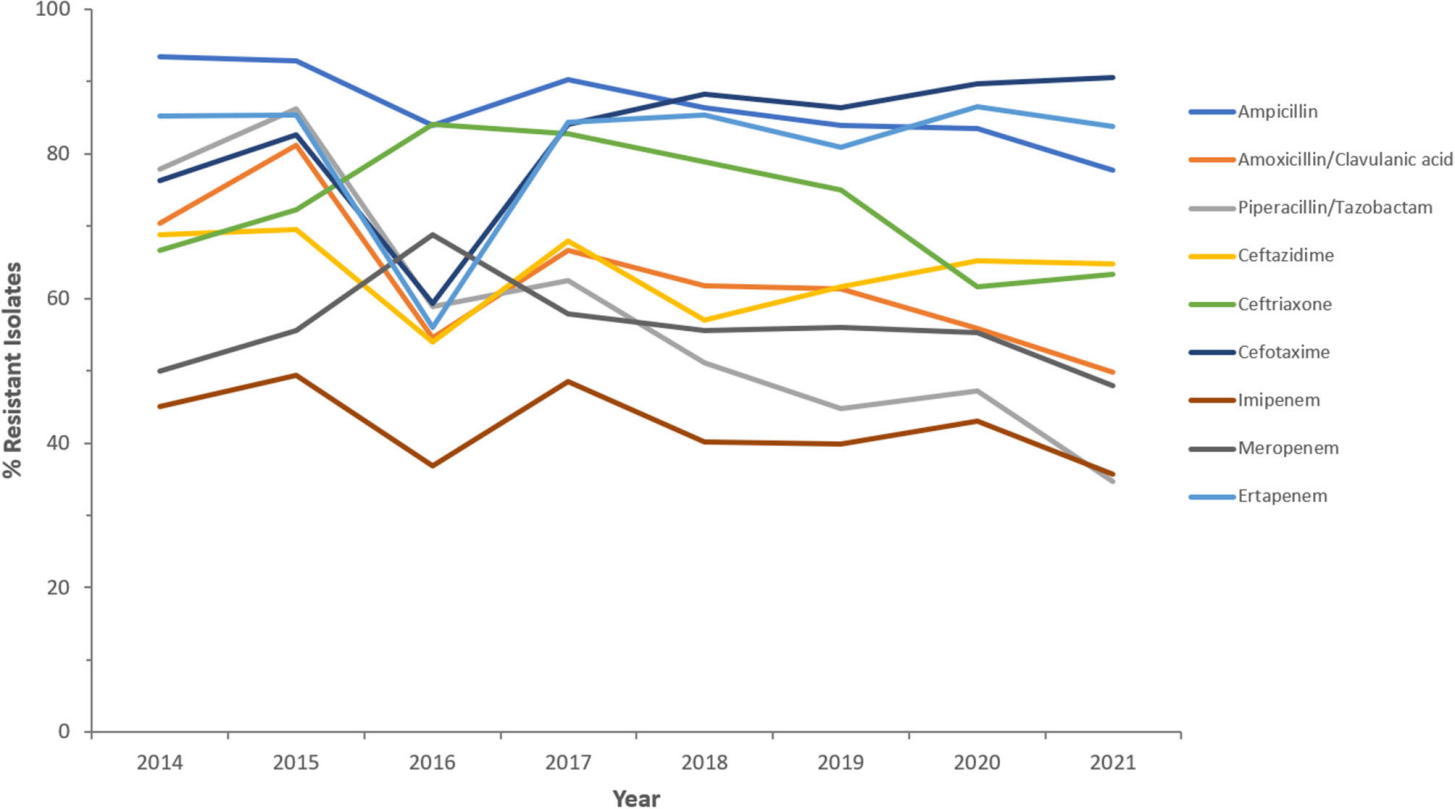
Resistance patterns (%R) to antibiotics of the β-lactam group among CREc for the period between 2014 and 2021. The graph shows a selected period due to small number of participating centers prior to 2014.

**Figure 8:**
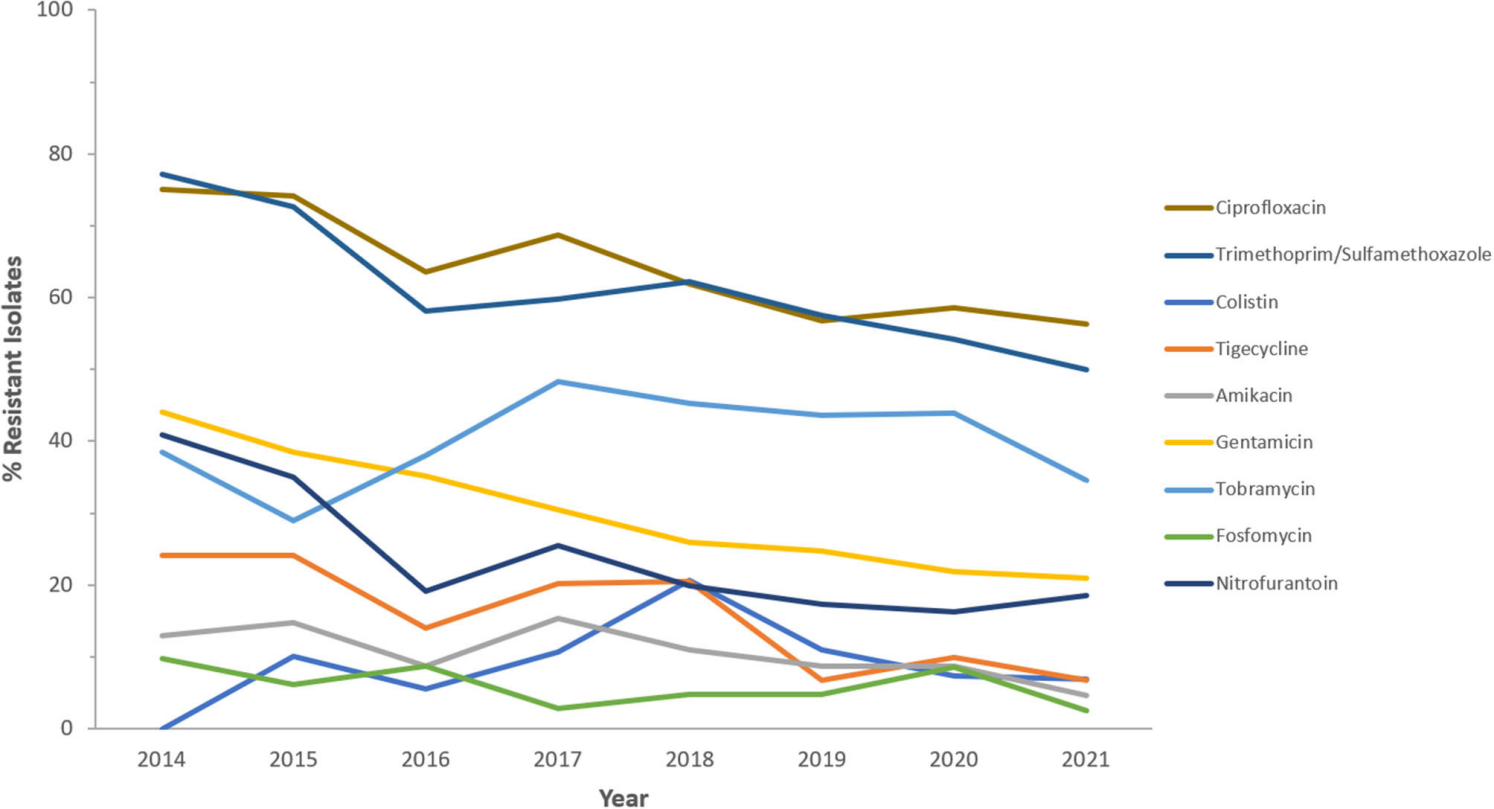
Resistance patterns (%R) to non β-lactam antibiotics among CREc for the period between 2014 and 2021. The graph shows a selected period due to small number of participating centers prior to 2014.

Resistance rates to cefotaxime increased from 87.2% (*n* = 179) in 2014 to 95.8% (*n* = 612) in 2021 for CRKp and from 76.3% (*n* = 59) in 2014 to 90.5% (*n* = 243) in 2021 for CREc. The trends of resistance in CRKp and CREc to different antibiotics for a selected time frame of the surveillance period are shown in Figures 5–8. It is noticed that the resistance to the antibiotics tested did not change much for CRKp over these years, while a more fluctuating pattern was seen for CREc, especially for cefotaxime and piperacillin/tazobactam. Regarding colistin, sensitivity fluctuated over the years but notably increased in CRKp after 2017, and it remained above 80% toward the end of the data collection period. Fosfomycin resistance levels were the lowest, with maximum upper limit of 8.6% in 2019 for CREc, while resistance levels for CRKp persisted close to zero with isolates remaining highly sensitive to this antibiotic all over the years.

### 3.10 Trend of carbapenem resistance during the surveillance period

Over the surveillance period, the resistance rates to individual carbapenems remained high, especially in CRKp, and in 2021 were equivalent to 67.6% for imipenem, 76.2% for meropenem, and 91.6% for ertapenem. Concerning CREc, resistance rates to meropenem and ertapenem were oscillating around 58 and 83% respectively over the 12 years, while for imipenem a progressive decrease was noted from 45.1% (*n* = 91) in 2014 to 35.6% (*n* = 932) in 2021.

Statistical analysis revealed a significant decreasing trend for imipenem and meropenem resistance in *Klebsiella species* (*p* < 0.01) while the decrease in ertapenem resistance was non-significant. Concerning *E. coli*, there was a statistically significant decreasing trend for meropenem and imipenem resistance over the 12 years while ertapenem resistance was associated to a statistically significant increasing trend with 83.8% of *E. coli* exhibiting ertapenem resistance in 2021.

A total of 1,002 CRKp and 982 CREc was tested for ceftolozane/tazobactam susceptibility and results revealed, respectively 65.6 and 57.9% resistance to this β-lactam/β-lactamase inhibitor combination. A total of 913 CRKp and 146 CREc was tested for ceftazidime/avibactam susceptibility and results revealed, respectively, 62.7 and 65.8% resistance to that second combination.

## 4 Discussion

This study was carried out to assess the contemporary trends of carbapenem resistance among *Enterobacterales* of medical relevance in the United Arab Emirates (UAE) over a 12-year period. The follow-up of CRE, characterized by mobile, easily transmissible resistance determinants, as well as easy spread facilitated by international travel and medical tourism, is imperative for infection surveillance and control in a country with huge cross-cultural exchange like the UAE. The participation of healthcare sites, both hospitals and clinics, in contribution to *Enterobacterales* data increased over the years from only 22 centers in the first year of reporting to more than 300 sites toward the end of the study period, representing the seven Emirates. This reflects not only the increasing coverage and representation of the surveillance database, but probably also the increased alertness across the country to the importance of antimicrobial resistance surveillance and mitigation.

The overall prevalence of CRE over the 12 years of surveillance averages at 3.8%. This result should be interpreted by comparison with figures of resistance obtained from similar follow-up studies that monitored carbapenem resistance for longitudinal periods, especially those from the region, due to patient and cultural exchange that connects these countries with the UAE. For example, in a surveillance from Africa and Middle East, a rate of 5.7% of resistance among *Enterobacterales* to carbapenems was reported (31). A report of antimicrobial resistance trends in Lebanon over 10 years, from 2000 till 2010, showed carbapenem resistance rates among *E. coli* and *K. pneumoniae* that did not exceed 2% (32). In a more recent analysis in 2022, the rate in Lebanon was 2.8% (33), while in Jordan, the rate was 1.6% in 2015 according to the Study for Monitoring Antimicrobial Resistance Trends (SMART) (34), and 1% in another study of 5 hospitals in 2018 (35). A version of the SMART study in Asia-Pacific region from 2002 to 2010 showed an overall carbapenem resistance rate of 10% among *Enterobacterales* (36). A recent surveillance report from Saudi Arabia, a neighboring country, showed resistance rates of about 5% to carbapenems among these bacteria (37). Another 5-year surveillance study from the Kingdom of Bahrain showed CRE average incidence of approximately 23/10,000 hospital admissions, with a decrease noted in the last two study years due to development and implementation of new CRE policy based on initial CRE screening for high risk patients, reinforcement of contact precautions, strengthened communication about CRE across hospital units, and staff education (38). As such, UAE, like other countries in the region, is facing the challenge of an important number of reported cases of CRE. Hence, update and follow-up on the prevalence, epidemiology and microbiological characteristics of CRE is mandatory for adequate public health and infection control practices.

Between 2013 and 2014, CRE prevalence increased from 1.7 to 3.7%, whereas from 2016 to 2020, CRE resistance prevalence shows a slightly decreasing pattern, which triggers the exploration of what factors resulted in such changes? In June 2013, the Health Authority of Abu Dhabi issued a circular on CRE, which has alerted healthcare facilities in the Emirate of Abu Dhabi and may have led to an increased detection of carbapenem-resistant pathogens, hence the increased prevalence of CRE in 2014. The number of surveillance sites in 2018 increased by 37 compared to 2017, but CRE prevalence in 2018 declined by 0.4 and 0.8% compared to 2017 and 2016, respectively. This decline cannot be directly explained from our results but warrants investigation of any national policies that may have produced such effect in these 2 years. In December 2017, the Department of Health of Abu Dhabi issued a standard and a guideline for antimicrobial stewardship (ASP), which may have contributed to improved prevention and control of multi-drug resistant organisms, including CRE, in the Emirate of Abu Dhabi. In 2019 and 2020, almost a steady pattern is observed, which tends to raise again in 2021, warranting to explore the effect of the COVID-19 pandemic on such changes. Previous data from other countries have reported a decline in CRE in the wake of the global pandemic (39, 40), and factors that may explain such decline like improved hygiene, social distancing, reduced travel, constricted transfer of critically ill patients, and others, have been described (41), although precise data in this regard remain conflicting (42). Moreover, the mortality rate, according to our observations, was about six-fold higher in patients associated with CRE compared to those associated with non-CRE *Enterobacterales*. Patients associated with CRE were four-fold more likely to be admitted to ICU, and their median length of stay was increased by 10 days, as compared to patients associated with non-CRE. This is consistent with other findings that indicated high mortality rate and poor outcomes in patients with CRE (43, 44), and highlights need for surveillance and control for better health outcomes.

When looking into the age of the population affected by CRE, it was found that over the study period, almost 90% of the patients with CRE samples were adults aged above 19 years. It is worth mentioning that the number of CRE isolates recovered from patients below 19 years increased from two isolates in the first year of the study to over 250 for CRKp and over 350 for CREc in the last year. Whether such an increase is due to resistance spread in the pediatric patients or merely due to increased inclusiveness of our samples by more centers getting involved, cannot be accurately determined, but indeed, warrants attention to monitor CRE in pediatrics. Although studies exist on CRE infections in pediatric patients (45–47), the true prevalence and proportionality to adult infections remains to be identified. One study reported that the frequency of carbapenem resistance among *Enterobacterales* in children in the United States raised from 0% in 1999–2000 to 0.47% in 2010–2011 (48). While the therapeutic paradigms for CRE have evolved with the introduction of novel β-lactam/β-lactamase inhibitor combinations like ceftazidime/avibactam, meropenem/vaborbactam, and imipenem/cilastatin-relebactam, optimal treatment of CRE infections in children remains challenging given limited pediatric-specific clinical data and experience (49). With the complexity of CRE treatment in children, and the need for expert consultation and individualized approach, our results call for a more meticulous surveillance of these infections in children while they are still limited, in a way to benefit from time until treatment paradigms evolve and new agents in the antibiotic pipeline become available and well-studied in pediatrics.

The majority of CRE identified throughout the study period were recovered from urine samples followed by sputum then blood. These data are somehow in alignment with other studies (50) but are unlike results of some large-scale multicenter studies from China and Taiwan reporting the highest number of CRE infections to originate in the lower respiratory tract (51–53). Urine samples may have outnumbered other samples in the UAE since most of the participating centers were public or private clinics rather than tertiary care centers, and these clinics may have urine as the easiest and most convenient sample. This highlights the possible community spread of these strains, that has been already reported elsewhere (6, 54), and warrants close monitoring in the UAE.

Throughout the study, CRKp remained the most prevalent CRE isolated from the studied samples, with an increase in its numbers consistently shown across the years. Pathogenic strains of *K. pneumoniae* cause widely diverse infectious diseases, including urinary tract, respiratory tract and blood infections, and are known as key menace to public health, being a common agent of nosocomial and community acquired infections (55). The results obtained regarding the demographic features of patients from whom CRKp isolates were recovered, together with antimicrobial susceptibility profiles, add to previous longitudinal data on CRKp in other countries over several years, like those from China (56–58), Singapore (59), Italy (60), and Germany (61). As a first time-trend study in the UAE, it will be beneficial to capitalize on these data for further surveillance of CRKp, and to try to associate its infections with particular risk factors. Our results did not reveal molecular epidemiology of the strains, a highly demanding task given the large number of samples and the long study period, but such properties, indeed, are tempting to analyse. So far, carbapenemase production, especially the Ambler class A *K. pneumoniae* carbapenemase (KPC) and the Ambler class B metallo-β-lactamases (MBL) like IMP, VIM, and NDM constitute the basic molecular mechanisms of CRKp emergence (12). According to recent evidence, knowledge of the exact mechanism of CRKp emergence is crucial to select an appropriate antimicrobial agent among choices such as plazomicin, eravacycline, temocillin, cefiderocol, ceftolozane/tazobactam, imipenem/cilastatin/relebactam, meropenem/vaborbactam, ceftazidime/avibactam, or aztreonam/avibactam (62). For instance, meropenem/vaborbactam combination is known for its effectiveness against KPC producers, ceftazidime/avibactam against both KPC and OXA-48 producers, and cefiderocol against MBL producers (63). It is anticipated that if resistance mechanism data support the phenotypic and demographic characteristics of CRKp, a better guide into antimicrobial therapy selection for these strains in the UAE can be established.

Regarding CREc and its isolation mostly from urine samples of outpatients, especially adult females, these are trends consistent with previously reported data about this organism (64–66). They may relate to its association with urinary tract infections (67, 68), which are among the most common infections worldwide, with substantial morbidity, mortality, and economic burden (69). Due to the physiological and structural factors, women are more vulnerable to urinary tract infections and almost half of them will experience at least one episode during their lifetime (70). In addition, the prevalence of the infection increases with age, weak immune system, and low estrogen levels (71). The high empiric use of antibiotics for the treatment of urinary tract infections has driven antibacterial resistance in *E. coli* (72), and this is not an exception in UAE, with its mixed and fluctuating population. Perhaps a more thorough investigation of carbapenem resistance in this organism by molecular and genomic methods will add to the available data from this 12-year long follow-up to better understand and mitigate carbapenem resistance in this organism.

Looking into the carbapenem resistance rates for specific CRE, it was noticed that in 2021, *K. pneumoniae* were to 67.6, 66.2, and 91.6% resistant to imipenem, meropenem, and ertapenem, respectively. There is a need to activate and reinforce stewardship programs and infection control to reduce further raise in carbapenem resistance in *K. pneumoniae* in the UAE. For imipenem, a progressive decrease in resistance among CREc was noted, reaching 35.6% in 2021, and is similar to some other studies describing trends in other areas in the region like Iraq and Jordan (73). This observation is interesting and emphasizes the effectiveness of infection control programs and the importance of targeted antimicrobial stewardship programs in reducing resistance rates.

**Figure 9:**
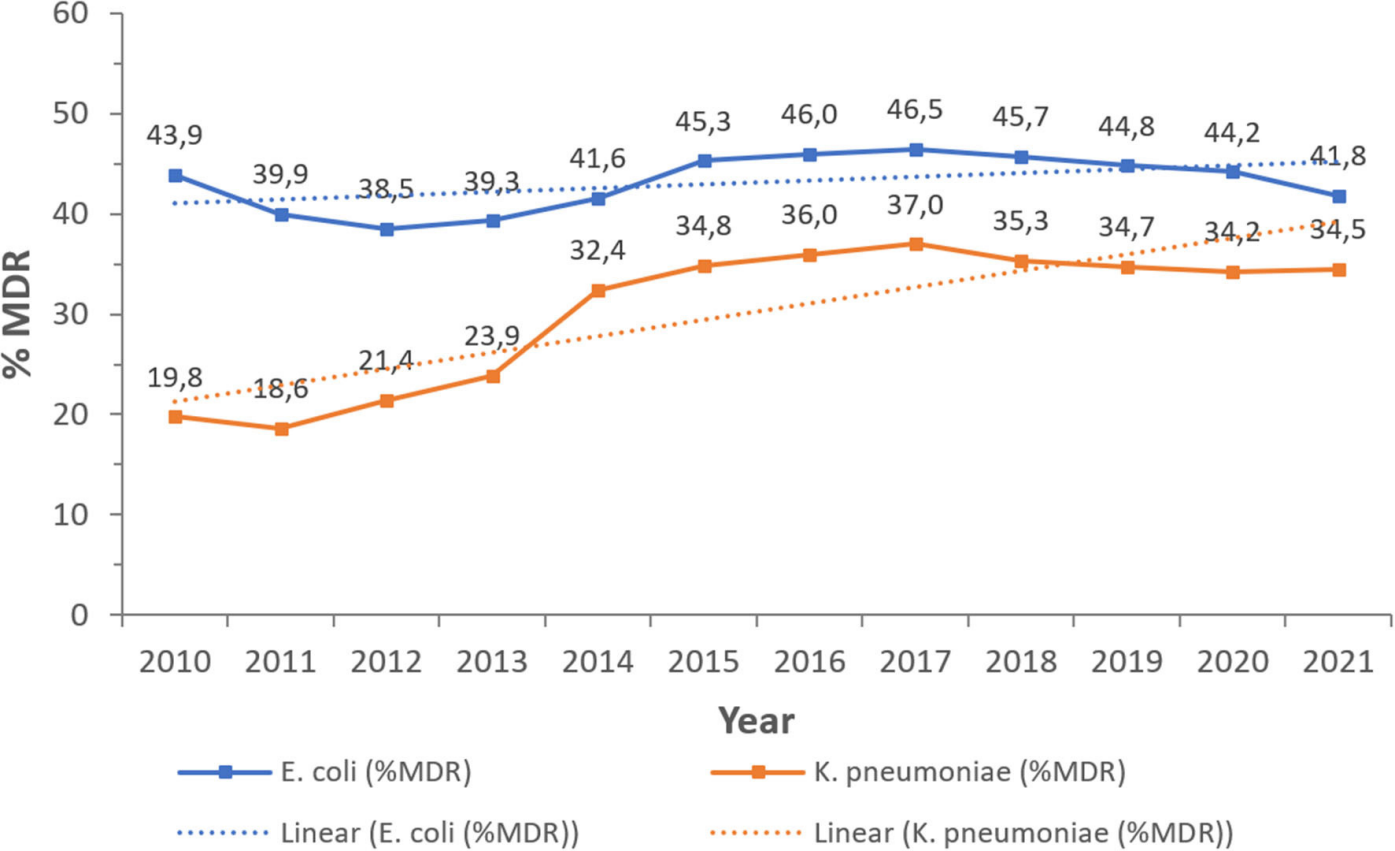
Percentages of CRKp and CREc over the surveillance period that have a MDR phenotype.

Apart from carbapenems, and for other antibiotic/antibiotic combinations tested throughout the study, resistance was high especially in CRKp, and showed a heterogeneous pattern for CREc. Nevertheless, both pathogens remained sensitive to fosfomycin, known for effectiveness in urinary tract infections caused by resistant *E. coli* and *K. pneumoniae* strains (74–76). However, in light of the recent observations of acquired fosfomycin resistance in these pathogens (77–79), practitioners in the UAE should remain vigilant about the use of this antibiotic to preserve its effectiveness. Moreover, CREc remained, throughout the study, sensitive to tigecycline, which persists among the last resort options for CRE (80, 81). Likewise, emerging reports of increased resistance in *E. coli* to this antibiotic (82, 83), as well as of hypervirulent *K. pneumoniae* which is tigecycline non-susceptible (84, 85) highlight the urgent need to enhance clinical awareness regarding this issue, the responsible use of tigecycline, and continuous epidemiologic surveillance to prevent compromising the usefulness of this antibiotic. Also, and with spread of mobilized colistin resistance genes (mcr) among Gram-negative pathogens (86) and reports from surrounding regions (87) as well as national observations (88), care must be taken to advance the knowledge about colistin resistance while supporting the efforts toward better stewardship to maintain clinical utility of this antibiotic. Moreover, the increase in MDR phenotype recovery in CRKp over the study years, being resistant to at least three antibiotic classes, indicates the need for follow-up, and both species need to be monitored in this regard, given the paucity of treatment options with multi-resistance (Figure 9).

## 5 Conclusion

In summary, this manuscript shows the trend over time of carbapenem resistance rates in the UAE among *Enterobacterales* and points out important findings for research and follow-up. It also shows that CRE infections are associated with higher mortality, increased ICU admission rates, and a longer hospitalization, thus poorer clinical outcome and higher associated costs. The phenotypic and demographic resistance profiles of CRE remain dynamic, and should be continuously updated, as well as supported by molecular epidemiology and genomic data, to help diminish the spread of these isolates across the UAE.

## Data availability statement

The datasets presented in this article are not readily available because the national AMR Surveillance database managed by the UAE Ministry of Health and Prevention (MOHAP) contains confidential health information. Requests to access the datasets should be directed to the UAE Ministry of Health and Prevention (https://mohap.gov.ae/).

## Ethics statement

Ethical approval for this study was provided by the Ministry of Health and Prevention Research Ethics Committee (MOHAP/DXB-REC/D.D.D/No.131/2021 and MOHAP/DXB-REC/J.J.J./No. 86/2023), Dubai Scientific Research Ethics Committee (DSREC-GL17-2023), and Abu Dhabi Health Research and Technology Ethics Committee (DOH/ZHCD/2023/1316).

## Author contributions

Conceptualization and data interpretation: CA, JT, AS, NA, GM, and DE. Data collection and manuscript review and editing: JT, CA, AS, NA, GM, DE, and The UAE AMR surveillance consortium. Formal analysis and manuscript preparation: JT and CA. All authors have read and agreed to the published version of the manuscript.

## Appendix

**Appendix 1:** ^‡^The UAE AMR Surveillance Consortium.

**Nr**	**Name**	**Institution**
1	Ahmed Elhag Ahmed	UAE University, College of Medicine and Health Sciences, Al Ain
2	Ahmed F. Yousef	Department of Biology, Center for Membranes and Advanced Water Technology, Khalifa University, Abu Dhabi
3	Amna AlBlooshi	Purelab, Al Ain
4	Dr. Adnan Alatoom	Sheikh Shakhbout Medical City (SSMC), Abu Dhabi
5	Dr. Ahmed Abdulkareem Al Hammadi	Tawam Hospital, Al Ain
6	Dr. Alaa MM Enshasy	Dubai Health Authority, Dubai
7	Dr. Amal Mubarak Madhi	Abu Dhabi Public Health Center, Abu Dhabi
8	Dr. Anju Nabi	Dubai Academic Health Corporation (DAHC), Dubai
9	Dr. Anup Shashikant Poddar	Al Sharq Hospital, Fujairah
10	Dr. Arun Kumar Jha	Danat Al Emarat Hospital, Abu Dhabi
11	Dr. Ayesha Abdulla Al Marzooqi	Abu Dhabi Public Health Center, Abu Dhabi
12	Dr. Bashir Aden	Khalifa University, Abu Dhabi
13	Dr. Deeba Jafri	Purelab, Sheikh Khalifa Medical City, Ajman
14	Dr. Duckjin Hong	Sheikh Khalifa Specialty Hospital (SKSH) RAK
15	Dr. Farah Ibrahim Al-Marzooq	United Arab Emirates University, Al Ain
16	Dr. Fatima Al Dhaheri	United Arab Emirates University, Al Ain
17	Dr. Ghada Abdel Wahab	Abu Dhabi Agriculture and Food Safety Authority, Abu Dhabi
18	Dr. Ghalia Abdul Khader Khoder	University of Sharjah, Sharjah
19	Dr. Gitanjali Avishkar Patil	NMC Specialty Hospital, Abu Dhabi
20	Dr. Hafiz Ahmad	RAK Hospital, Ras Al Khaimah
21	Dr. Hazim Khalifa	Department of Veterinary Medicine, UAE University, Al Ain
22	Dr. Husein Alzabi	Sheikh Khalifa General Hospital, Um al Quwain
23	Dr. Ibrahim Alsayed Mustafa Alhashami	Purelab, Al Qassimi Hospital, Sharjah
24	Dr. Irfaan Akthar	Mediclinic City Hospital, Dubai
25	Dr. Jens Thomsen	Abu Dhabi Public Health Center, Abu Dhabi
26	Dr. John Stelling	WHONET, Boston, USA
27	Dr. Kavita Diddi	Prime Hospital, Dubai
28	Dr. Krishnaprasad Ramabhadran	Burjeel Hospital, Abu Dhabi
29	Dr. Laila Al Dabal	Dubai Academic Health Corporation (DAHC, Dubai)
30	Dr. Madikay Senghore	Khalifa University, Abu Dhabi
31	Dr. Manal Abdel Fattah Ahmed	PureLab, Ras Al Khaimah
32	Dr. Maya Habous	Rashid Hospital, Dubai Academic Health Corporation, Dubai
33	Dr. Moeena Zain	American Hospital Dubai
34	Dr. Monika Maheshwari	Al Zahra Hospital, Dubai
35	Dr. Monika Maheshwari	Medeor 24x7 Hospital, Dubai
36	Dr. Mubarak Saif Alfaresi	Zayed Military Hospital, Abu Dhabi
37	Dr. Mushtaq Khan	United Arab Emirates University, Al Ain
38	Dr. Najiba Abdulrazzaq	Al Kuwait Hospital, Emirates Health Services Establishment, Dubai
39	Dr. Nehad Nabeel Al Shirawi	Al Fujairah Hospital
40	Dr. Nesrin Helmy	Mediclinic Al Noor Hospital - Khalifa Street, Abu Dhabi
41	Dr. Prashant Nasa	NMC Specialty Hospital Al Nahda, Dubai
42	Dr. Rajeshwari T. A. Patil	Burjeel Medical City, Abu Dhabi
43	Dr. Ratna A. Kurahatti	NMC Royal Hospital Khalifa City A, Abu Dhabi
44	Dr. Riyaz Amirali Husain	Dubai Hospital, Dubai Academic Health Corporation, Dubai
45	Dr. Robert Lodu Serafino Wani Swaka	Sheikh Shakhbout Medical City, Abu Dhabi
46	Dr. Savitha Mudalagiriyappa	University Hospital Sharjah, Sharjah
47	Dr. Seema Oommen	Burjeel Medical City, Abu Dhabi
48	Dr. Shaikha Ghannam Alkaabi	Abu Dhabi Public Health Center, Abu Dhabi
49	Dr. Simantini Jog	Fakeeh University Hospital, Dubai
50	Dr. Simantini Jog	King's College Hospital London Dubai Hills, Dubai
51	Dr. Siobhan O‘Sullivan	Khalifa University, Abu Dhabi
52	Dr. Somansu Basu	NMC Specialty Hospital, Al Ain
53	Dr. Yassir Mohammed Eltahir Ali	Animal Wealth Sector, Abu Dhabi Agriculture and Food Safety Authority, Abu Dhabi
54	Dr. Yousuf Mustafa Naqvi	Department of Health Abu Dhabi (DoH), Abu Dhabi
55	Dr. Zulfa Omar Al Deesi	Latifa Maternity & Pediatric Hospital, Dubai
56	Emmanuel Fru Nsutebu	Sheikh Shakhbout Medical City, Abu Dhabi
57	Fouzia Jabeen	Purelab, Sheikh Khalifa Hospital, Abu Dhabi
58	Francis Amirtharaj Selvaraj	Sheikh Khalifa Medical City (SKMC), Abu Dhabi
59	Hadayatullah Ghulam Muhammad	Emirates International Hospital, Al Ain
60	Imene Lazreg	University of Sharjah, Sharjah
61	Kaltham Ali Kayaf	Ministry of Climate Change & Environment (MOCCAE), Dubai
62	Laura Thomsen	University of Freiburg, Germany
63	Leili Chamani-Tabriz	Clemenceau Medical Center, Dubai
64	Pamela Fares Mrad	Abu Dhabi Public Health Center (ADPHC), Abu Dhabi
65	Pascal Frey	Berne University Hospital, Berne, Switzerland
66	Prof. Abiola Senok	College of Medicine, Mohammed Bin Rashid University of Medicine and Health Sciences, Dubai
67	Prof. Agnes-Sonnevend-Pal	University of Pécs, Pécs, Hungary
68	Prof. Andreas Podbielski	University Hospital Rostock, Rostock, Germany
69	Prof. Carole Ayoub Moubareck	College of Natural and Health Sciences, Zayed University, Dubai
70	Prof. Dean Everett	Department of Pathology and Infectious Diseases, College of Medicine, Khalifa University, Abu Dhabi
71	Prof. Godfred A. Menezes	Department of Medical Microbiology and Immunology, RAK Medical and Health Sciences University, Ras Al Khaimah
72	Prof. Hala Ahmed Fouad Ismail	PureLab, Al Qassimi Hospital, Sharjah
73	Prof. Mohamud M. Sheek-Hussein	United Arab Emirates University, Al Ain
74	Prof. Peter Nyasulu	Department of Global Health, Faculty of Medicine and Health Sciences, Stellenbosch University, South Africa
75	Prof. Sameh Soliman	University of Sharjah, Sharjah
76	Prof. Tibor Pal	University of Pécs, Pécs, Hungary
77	Rania El Lababidi	Dept. of Pharmacy Services, Cleveland Clinic Abu Dhabi
78	Saeed Hussein	Erada Center for Treatment and Rehabilitation, Dubai
79	Stefan Weber	Purelab, Abu Dhabi
80	Sura Khamees Majeed	Al Gharbia Hospitals - Madinat Zayed Hospital
81	Syed Irfan Hussein Rizvi	Mediclinic City Hospital, Dubai
82	Timothy Anthony Collyns	Tawam Hospital, Al Ain
83	Zahir Osman Babiker	Sheikh Shakhbout Medical City (SSMC), Abu Dhabi
